# Fine mapping of barley locus *Rps6* conferring resistance to wheat stripe rust

**DOI:** 10.1007/s00122-015-2663-1

**Published:** 2016-02-13

**Authors:** Kun Li, Joshua Hegarty, Chaozhong Zhang, Anmin Wan, Jiajie Wu, Gina Brown Guedira, Xianming Chen, María Muñoz-Amatriaín, Daolin Fu, Jorge Dubcovsky

**Affiliations:** State Key Laboratory of Crop Biology, Shandong Key Laboratory of Crop Biology, Shandong Agricultural University, Tai’an, 271018 Shandong China; Department of Plant Sciences, University of California, Davis, CA 95616 USA; Department of Plant Pathology, Washington State University, Pullman, WA 99164 USA; USDA-ARS, Plant Science Research Unit, Department of Crop Science, North Carolina State University, Raleigh, NC 27695 USA; USDA-ARS, Wheat Genetics, Quality, Physiology, and Disease Research Unit, Pullman, WA 99164 USA; Department of Botany and Plant Sciences, University of California, Riverside, CA 92521 USA; Howard Hughes Medical Institute, Chevy Chase, MD 20815 USA

## Abstract

*****Key message***:**

**Barley resistance to wheat stripe rust has remained effective for a long time and, therefore, the genes underlying this resistance can be a valuable tool to engineer durable resistance in wheat.**

**Abstract:**

Wheat stripe rust, caused by *Puccinia striiformis* f. sp. *tritici* (*Pst*), is a major disease of wheat that is causing large economic losses in many wheat-growing regions of the world. Deployment of *Pst* resistance genes has been an effective strategy for controlling this pathogen, but many of these genes have been defeated by new *Pst* races. In contrast, genes providing resistance to this wheat pathogen in other grass species (nonhost resistance) have been more durable. Barley varieties (*Hordeum vulgare* ssp. *vulgare*) are predominately immune to wheat *Pst*, but we identified three accessions of wild barley (*Hordeum vulgare* ssp. *spontaneum*) that are susceptible to *Pst*. Using these accessions, we mapped a barley locus conferring resistance to *Pst* on the distal region of chromosome arm 7HL and designated it as *Rps6*. The detection of the same locus in the cultivated barley ‘Tamalpais’ and in the Chinese barley ‘Y12’ by an allelism test suggests that *Rps6* may be a frequent component of barley intermediate host resistance to *Pst*. Using a high-density mapping population (>10,000 gametes) we precisely mapped *Rps6* within a 0.14 cM region (~500 kb contig) that is colinear to regions in *Brachypodium* (<94 kb) and rice (<9 kb). Since no strong candidate gene was identified in these colinear regions, a dedicated positional cloning effort in barley will be required to identify *Rps6*. The identification of this and other barley genes conferring resistance to *Pst* can contribute to our understanding of the mechanisms for durable resistance against this devastating wheat pathogen.

## Introduction

Although more than 700 million tons of wheat (*Triticum* spp.) are produced per year worldwide (FAOSTAT 2013), further increases are required to support a growing human population. An important component of these increases in global production is the reduction of yield losses caused by various wheat pathogens. Wheat stripe rust, caused by *Puccinia striiformis* Westend. f. sp. *tritici* Erikss. (*Pst*), is one of the most destructive fungal diseases and is causing substantial yield and quality losses in many of the wheat-growing regions of the world (Chen et al. 2014; Wellings 2011). The appearance and spread of more virulent and aggressive *Pst* races since the beginning of this century has exacerbated the problem (Hovmøller et al. 2010; Wan and Chen 2014).

Fungicides can be applied to control *Pst*, but they generate additional costs and are potentially harmful to the environment. In contrast, the deployment of genetic sources of *Pst* resistance is a reliable, environmentally friendly, and cost effective alternative to control *Pst*. However, the rapid evolution of novel *Pst* races has rendered many of these resistance genes ineffective (Chen et al. 2010), and has prompted the search for more durable sources of *Pst* resistance.

A possible path to a more durable resistance is the identification and deployment of genes conferring resistance to *Pst* from plant species that are not normal hosts of this pathogen. This type of resistance is usually referred to as “nonhost resistance”. Most pathogens cannot infect and cause disease on species that are different from their normal hosts, and this nonhost resistance is usually effective over long evolutionary periods (Bettgenhaeuser et al. 2014). However, the effectiveness and underlying genetic complexity of nonhost resistance is usually correlated with the taxonomic distance among the host and nonhost species (Bettgenhaeuser et al. 2014).

Studies of Arabidopsis resistance to the barley powdery mildew pathogen (*Blumeria graminis* f. sp. *hordei*, henceforth *Bgh*) provide a good example of nonhost resistance against a pathogen from a very distantly related host species. Screenings of Arabidopsis mutants with increased *Bgh* penetration and haustoria formation (*PEN* genes) identified resistance genes *PEN1*, *PEN2*, and *PEN3* (Collins et al. 2003; Lipka et al. 2005; Stein et al. 2006). However, even the Arabidopsis plants carrying all three mutations remained resistant to *Bgh* (Johansson et al. 2014). These results suggest that Arabidopsis nonhost resistance to pathogens from very distantly related species may have a complex genetic basis.

In contrast, barley resistance to *Pst* is not as effective as Arabidopsis resistance to *Bgh*, and seems to have a simpler genetic basis. Races of *Pst* usually do not infect barley, and those that infect barley (*P. striiformis* f. sp. *hordei* Erikss., henceforth *Psh*) are not often virulent on wheat. However, there are barley genotypes that can be infected by some *Pst* races and some wheat genotypes that can be infected by some *Psh* races (Chen et al. 1995; Kumar et al. 2012; Niks 1987; Pahalawatta and Chen 2005; Sui et al. 2010). In addition, two genetic studies have shown that barley resistance to *Pst* is determined mainly by few major genes (Pahalawatta and Chen 2005; Sui et al. 2010). These characteristics suggest that wheat and barley are in the earlier stages of development of nonhost resistance to different formae speciales of *P. striiformis*. The terms “intermediate host” and “intermediate non-host” resistance have been proposed to accommodate the continuum of rust infection outcomes observed in the transition from host to nonhost resistance (Bettgenhaeuser et al. 2014). Based on the characteristics described above, barley resistance to *Pst* can be classified as “intermediate host resistance”.

Unfortunately, none of the genes underlying barley resistance to *Pst* has been identified so far, limiting our ability to test the effectiveness and durability of barley intermediate host resistance genes transferred to wheat. As a first step in the identification of barley genes conferring resistance to *Pst*, we developed a high-density map for a *Pst* resistance locus on chromosome 7H and explored the colinear regions in the rice and *Brachypodium* genomes for candidate genes. We also tested the presence of this resistance locus in the cultivated barley variety Tamalpais. The long-term objective of this project is to understand the genetic basis of barley intermediate host resistance to *Pst* and to use that knowledge to generate wheat lines with more durable resistance to *Pst.*

## Materials and methods

### Plant materials

In 2010, 32 accessions of *Hordeum vulgare* ssp. *spontaneum* (K. Koch) Thell were screened in the field for resistance to *Pst* at the University of California, Davis (henceforth, UCD field). Two susceptible accessions (PI 264220 and PI 560559) collected in Turkey and two *Pst* resistant accessions (PI 466050 and PI 466186) collected in Syria (Table 1) were selected to develop two F_2_ populations segregating for *Pst* resistance. The first population, generated from the cross PI 466050 × PI 264220, was designated as POP366 and included 127 F_2_ plants. The second population, generated from the cross PI 466186 × PI 560559, was designated as POP371 and included 132 F_2_ plants.

**Table 1:** Reaction of *Hordeum vulgare* ssp. *spontaneum* to *Puccinia striiformis* f. sp. *tritici* in the UCD field in 2010

Accession no.	Origin	*Pst* reaction
PI 236386	Syria	Resistant
PI 244772	Pakistan	Resistant
PI 245740	Turkey	Resistant
PI 264220	Turkey	Susceptible
PI 282583	Israel	Resistant
PI 282586	Israel	Resistant
PI 284757	Israel	Resistant
PI 293394	Turkmenistan	Susceptible
PI 293401	Turkmenistan	Resistant
PI 293402	Turkmenistan	Resistant
PI 293413	Azerbaijan	Resistant
PI 293414	Azerbaijan	Resistant
PI 296803	Israel	Resistant
PI 296814	Israel	Resistant
PI 405294	Israel	Resistant
PI 405295	Israel	Resistant
PI 405304	Israel	Resistant
PI 405346	Israel	Resistant
PI 466020	Syria	Resistant
PI 466033	Syria	Resistant
PI 466039	Syria	Resistant
PI 466049	Syria	Resistant
PI 466050	Syria	Resistant
PI 466058	Syria	Resistant
PI 466062	Syria	Resistant
PI 466157	Syria	Resistant
PI 466186	Syria	Resistant
PI 466249	Lebanon	Resistant
PI 466253	Lebanon	Resistant
PI 466673	Turkey	Resistant
PI 560558	Turkey	Resistant
PI 560559	Turkey	Susceptible

Since the same locus was identified in both populations, we focused on POP366 to develop a high-density map. From this population, we selected 24 F_2_ plants heterozygous for the two markers flanking the major *Pst* resistance locus, allowed them to self-pollinate, and produced abundant F_3_ seeds. We genotyped 5444 F_3_ plants (10,888 segregating chromosomes), identified 746 plants carrying recombination events between the flanking markers, and used them to generate a high-density map. Once the locus was mapped more precisely, we developed closer flanking markers and reduced the number of F_3_ plants carrying recombination events in the critical region to 129. Each of these 129 F_3_ plants was self-pollinated and the corresponding F_4_ families were evaluated for resistance to *Pst* to infer the genotype of the parental F_3_ plant.

Since the *Pst* resistance locus identified in this study mapped to a similar chromosome location as the *YrpstY1* locus in Chinese barley line ‘Y12’ (Sui et al. 2010), we performed an allelism test to determine if they were the same or different genes. We reciprocally crossed the *Pst* resistant lines Y12 and PI 466050 and generated an F_2_ population of 390 plants. This population was evaluated for susceptibility to *Pst* in 2015 at the UCD field facilities (*Pst* races used in the field inoculation are described below).

To determine if the locus identified in wild barley populations POP366 and POP371 was also present in cultivated barley (*H. vulgare* ssp. *vulgare*), we crossed the *Pst* resistant barley variety ‘Tamalpais’ (PI 645477, from California, used as male) with the *Pst* susceptible *H. vulgare* ssp. *spontaneum* accessions PI 264220 and PI 293394 (Table 1). The F_2_ lines were advanced to F_5_ by single-seed descent resulting in 161 lines that were tested for *Pst* resistance and were genotyped for markers linked to the resistance gene identified in POP366 and POP371.

### Tests of stripe rust reactions

The parental lines of POP366 and POP371 were tested at Washington State University (WSU) for their responses to four North American *Pst* races (PSTv-14, PSTv-37, PSTv-40, and PSTv-51) and two North American *Psh* races (PSH-48 and PSH-58). The seedling tests for stripe rust resistance were performed twice and produced consistent results.

The F_2_ populations of POP366 and POP371 were initially tested for their responses to *Pst* in 2011 at the Shandong Agricultural University in Tai’an, China (SDAU). To validate the phenotype of the F_2_ plants, F_3_ progeny tests were conducted in the same field in 2012. For the Tamalpais-related populations, single-seed descent (SSD) F_5_ lines were tested at SDAU in 2014. Since no natural infections of *Pst* or *Psh* occur in this region, studies at SDAU used artificial *Pst* inoculations. Due to changes in spore availability, different *Pst* races were used for the field inoculations in different years (2011: mixture of SY11, CYR31 and CYR32; 2012: CYR32, and 2014: mixture of CYR29, CYR31, CYR32 and CYR33). These have been predominant *Pst* races in China since the early 1990s, and their virulence profiles have been previously described (Wan et al. 2004; Chen et al. 2009). The 129 F_3_ plants carrying the critical recombination events were evaluated for resistance to *Pst* (some in growth chambers and others in the UCD field in 2014) and their F_4_ progeny were evaluated in the UCD field in 2015. For the 2015 experiment in the UCD field, plants were inoculated with a mixture of *Pst* urediniospores collected in the UCD field in 2014 from *Pst* susceptible wheat plants. Analysis of 24 *Pst* samples from infected leaves collected at the UCD field in 2014 indicated the presence of the following races (followed by their frequency in parentheses): PSTv-4 (4.2 %), PSTv-11 (4.2 %), PSTv-15 (8.3 %), PSTv-17 (8.3 %), PSTv-37 (25.0 %), PSTv-52 (41.7 %), and PSTv-53 (8.3 %). No differences in the reactions of the susceptible and resistant barley parental lines were observed among the field studies in China and the USA. This is not an unexpected result as most of the cultivated barely accessions are resistant to all *Pst* races (Chen et al. 1995).

For the growth chamber tests, plants were inoculated at the two-leaf stage with urediniospores and kept in a dark dew chamber at 10 °C for 24 h and then transferred to a growth chamber with a diurnal temperature cycle that changed gradually from 4 to 20 °C with 16 h photoperiod (Chen et al. 2002; Pahalawatta and Chen 2005). Infection types (ITs) were recorded 20–22 days after inoculation using the McNeal’s 0–9 scale reported before (Line and Qayoum 1992). To convert the *Pst* reactions into two genotypic classes for mapping purposes, IT scores from 0 to 4 were considered as resistant and IT scores from 6 to 9 as susceptible (plants with scores = 5 were not used in the classification).

### Genotyping, linkage mapping and QTL analysis

Genomic DNA was extracted from leaf tissues using the Sarkosyl method (Yuan et al. 2012), measured using ND-1000 spectrophotometry (Thermo Fisher Scientific, Wilmington, DE, USA), and normalized to 50 ng µl^−1^. A total of 93 F_2_ plants from POP371 and the two parental lines were genotyped using an Illumina VeraCode custom assay (del Blanco et al. 2014). This assay includes 384 single nucleotide polymorphisms (SNP) selected from the Illumina GoldenGate BOPA1 and BOPA2 assays for even coverage of the barley genome (Close et al. 2009). A genetic linkage map was created using the maximum likelihood mapping algorithm with the Kosambi function as implemented in JoinMap 4.0 (Kyazma B.V., Wageningen, Netherlands). The Windows QTL Cartographer V2.5 (Wang et al. 2012) was used to identify QTL for *Pst* resistance using composite interval mapping (window size: 10 cM; walk speed: 1 cM). Significance thresholds were established using 1000 permutation tests. QTL with a logarithm of odds (LOD) score of three or more were considered significant.

The degree of dominance was calculated using the formula: *D* = (2*X*_2_ − *X*_1_ − *X*_3_)/(*X*_1_ − *X*_3_) (Falconer 1964), where *X*_1,_*X*_2_ and *X*_3_ are the infection types scores of the plants homozygous for the markers flanking the *Rps6* resistant allele, the heterozygous, and the plants homozygous for the markers flanking the susceptible allele, respectively.

### Marker development

To increase marker density in the target region, the parents of POP366 and POP371 were genotyped with the 9 K barley iSelect platform and PCR markers were designed for SNP previously mapped between flanking markers *11_10885* and *11_11012* (Comadran et al. 2012). In addition, we utilized colinear regions in *Aegilops tauschii* (Luo et al. 2013) (http://aegilops.wheat.ucdavis.edu/ATGSP/), *Brachypodium*, and rice to identify candidate genes within the region. We then used the barley draft genome sequence (International Barley Genome Sequencing Consortium 2012) and sequences from gene-bearing BACs (Muñoz-Amatriaín et al. 2015) to develop additional markers. SNP and/or InDels were identified between the parents of POP366 and POP371 and PCR markers (e.g. CAP and dCAP) were developed. PCR primers, restriction enzymes and size of the expected products are described in Table 2. PCR products were separated in 6 % non-denaturing acrylamide or 2 % agarose gels.

**Table 2:** PCR markers used to map the *Pst* resistance locus in wild barley

Gramene ID^a^	Genbank ID	Locus ID^b^	Types^c^	Forward primers (5′→3′)	Reverse primers (5′→3′)	Enzymes^d^	Bands in bp^e^
*MLOC_36989*	AK358025	*11_10885*	InDel	TCTGCTCAGCAAGAAGAACG	AGCAATATTTACGCCGAACC	–	268 (431)
*MLOC_51298*	AK376744	*11_20847*	CAP	AACGTTGTGGCCTTTTATGG	TTCCAGTGTCGACGGAAGAT	*Hha*I	764 (465)
*MLOC_39511*	AK359663	–	InDel	GCTACAACAGTTGGCAAGTCTG	GAAAAGTGATCCGCGTGTTT	–	140 (131)
*MLOC_26380*	AK374153	–	CAP	CCACCTAACCTTGTGCCTTG	TGCTGCATTCCCATGTAAAC	*Afl*II	793 (1191)
*MLOC_26380* ^f^		Tamalpais	CAP	ATCTTTGGCCTGTTTGGTGT	TTAAGTGTCGACGGTGAACG	*TaqI*	698 (857)
*MLOC_16765*	AK366838	*11_10687*	CAP	TCGGTGAATCTGGGCTATTC	TCAGTGCACCAGTTCTTTCC	*Dde*I	326 (507)
*MLOC_52705*	AK374563	–	CAP	GTGCAAGCTGTCGTATGCTC	CCAACCGGCAAATGTTGAT	*Fok*I	578 (446)
*MLOC_18254*	CB880323	–	InDel	CCCCAAAACACCCTCAGGTCT	GAGGGACGCGGGGAAGCAAA	–	101 (107)
*Mx*-*contig_58199*	CAJW010058199	–	CAP	GGGGCTTCAGAGCATATCAG	GCCGGTGAAGTTACATTGCT	*NheI*	1180 (1327)
*MLOC_37425*	–	–	dCAP	CAGCCTTGTCACCGGAGAAGTAGTA	CATGTTTTTGGCCTTCACACA	*RsaI*	269 (295)
*MLOC_65262*	–	–	CAP	TTTCAATAGAAACACGCTCACA	CACACGCTTTCATCATCACC	*TaqI*	812 (510)
*MLOC_52532*	AK361699		dCAP	TGATTTAGCAGAGGAGGTTAC	GAACATACTCGCAAAGACTTGGT	*BsrI*	193(217)
*MLOC_37646*	AK362947	–	CAP	CTTGCACTTGTAAGGGCTGA	CTGGTTTTCAAACAGCAGCA	*Alu*I	366 (471)
*MLOC_37646* ^f^		Tamalpais	dCAP	ATCTGTCAAAGCCAAGTATTTGGTGATT	TCCAAAGCAATCACAGACG	*Hinf*I	293 (259)
*MLOC_22197*	AK250823	–	CAP	GAGACAGTCATCCCGGAGAG	CCGACAACCAGTTCAAGGTC	*Pvu*II	777 (1265)
*MLOC_24177*	AK363137	–	InDel	TGATGTGTCCACTTGCAAAAA	TAAGTGTGTGGTGCCTGGTG	–	190 (178)
*MLOC_6480*	AB032839	–	dCAP	GTAGTTGCTGTAATCAACATGGT	CAATGGCAAGACCAGTAGCA	*Hph*I	243 (263)
*MLOC_4670*	AK359069	*11_20139*	InDel	AGCTTGATGACCTTTCTGCAA	CACGAAGCGCTCAACACTAC	–	190 (179)
*MLOC_13779*	AK360979	*11_21223*	InDel	GACAACGTGGTGTTCCACAA	AACACGACGTCAGAACACAAGAGC	–	175 (144)
*MLOC_55101*	AK355501	*12_30593*	CAP	TGAGACTTTGTAATGGTGCCAA	TCTGTGAAACGCCTGCTAGAT	*Taq*I	460 (784)
*MLOC_71862*	AK250063	*11_20414*	CAP	ATTTGGGAACGGAGGGAATA	ATCTGCAGCGCGTAGTTGT	*Ava*II	861 (607)
*MLOC_75180*	AK252062	*11_11012*	InDel	TCGCCCAGGACAGCGACGTAA	GTTGAACCCGCTCTCCATC	–	166 (180)
*MLOC_36989*	AK358025	*11_10885*	CAP	CAGGAAGAGGCTCTCCAAGA	CAGAAACTCAGTGGCGATCA	*Ava*II	928 (782)
*MLOC_75180*	AK252062	*11_11012*	CAP	GAAGATCATGCAGGCACAGA	ATCACATTTCCAGTCCAACA	*Hpy99*I	545 (318)

^a^The first 20 markers were used for POP366 and the last 2 for POP371

^b^The locus ID in the 9 K barley iSelect chip (Comadran et al. 2012)

^c^ *CAP* cleavage amplification polymorphism, *dCAP* degenerate cleavage amplification polymorphism, *InDel* insertion/deletion

^d^Restriction enzymes used to digest the PCR product

^e^Size of PCR bands: the first number indicates the product from the resistant parent and the second number in parenthesis the product of the susceptible parent

^f^Different, SNP, PCR primers and restriction enzymes used in the Tamalpais-derived populations

To map barley loci (MLOC sequences from cultivar Morex) to barley FPC contigs, we blasted sequences of the mapped Morex loci against the Morex BAC end sequence database at IPK-Gatersleben. Only matches showing 100 % identity over more than 500 bp were considered as correct matches. Barley genome 082214v1 was used to establish the approximate position of the *Rps6* region on the 7H pseudomolecule (http://plants.ensembl.org/Hordeum_vulgare/Info/Index).

### Expression of genes linked to marker development

The expression of two genes completely linked to the resistance phenotype was studied in different tissues of the barley variety Golden Promise by RT-PCR. For gene MLOC_65262 we used forward primer 5′-TCGAGAGGCAGATCCAAGAT-3′ and reverse primer 5′-TTTTGGCAAACCACTCTCCT-3 (expected size of RT-PCR product 137 bp). For MLOC_37425 we used forward primer 5′-ATCGGAGAAGGAGGAGAATATGG-3′ and reverse primer 5′-TCATTTCAGAGGGTAAACAGCT-3′ (expected size of RT-PCR product 546 bp). *ACTIN* (expected size of RT-PCR product 692 bp) was used as endogenous control using primers described before (Abu-Romman et al. 2011).

The RT-PCR conditions included an initial denaturation step (94 °C 5 min), followed by 40 cycles of denaturation (94 °C, 30 s), annealing (58 °C, 30 s) and extension (72 °C, 1 min), and a final extension step (72 °C, 10 min). RNA was extracted from the middle region of leaf blades and sheaths and from roots of Golden Promise barley plants at the three-leaf stage, and from spikes before anthesis. Expression of these two genes was also explored in BARLEX (the Barley Draft Genome Explorer, Colmsee et al. 2015), where expression levels from RNAseq experiments in eight tissues from barley variety Morex are reported in a graphical form in FPKM (fragments per kb of exon per million reads mapped).

## Results

### Wild barley accessions show differential responses to *Pst* races

Three out of the 32 accessions of *H. vulgare* ssp. *spontaneum* that were evaluated in the UCD field in 2010 for *Pst* resistance, were found to be susceptible to *Pst* supporting abundant sporulation and the rest were resistant and did not support sporulation (Table 1). The seedling responses to *Pst* under controlled environmental conditions of the four *H. vulgare* ssp. *spontaneum* accessions selected as parental lines were consistent with the results observed in the field (Table 3). All four *H. vulgare* ssp. *spontaneum* accessions were highly susceptible to *Psh* races PSH-48 and PSH-58 but differed in their susceptibility to the four tested races of *Pst* (Table 3). Accessions PI 466050 and PI 466186 exhibited resistant responses to all *Pst* races (IT, 0–4) whereas accessions PI 264220 and PI 560559 were susceptible (IT, 6–8) to the same races. The cultivated barley control ‘Steptoe’ was susceptible to both *Psh* races and resistant to all four *Pst* races, whereas the wheat control ‘Avocet S’ was susceptible to all *Pst* races and resistant to both *Psh* races (Table 3).

**Table 3:** Reaction of *Hordeum vulgare* ssp. *spontaneum* seedlings to *Pst* and *Psh* races under controlled environments

Lines	*Pst* races				*Psh* races	
	PSTv-14 (PST-127 PST-139)^a^ 2009^b^	PSTv-37 (PST-100 PST-102) 2003	PSTv-40 (PST-114 PST-116) 2004–2005	PSTv-51 (PST-114+ PST-127) 2004–2007	PSH-48 2011	PSH-58 2001
PI 466050	1	1	1	1	7–8	8
PI 466186	1–4	1–4	1–4	1	7–8	8
PI 264220	5–8	8	7–8	8	8	7–8
PI 560559	5–7	7	7	6	7–8	8
Avocet S^c^	8	8	8	8	1	1
Steptoe^d^	1	1	1	1	7–8	8

^a^Names in parenthesis indicate similar races in the previous nomenclature system (Wan and Chen 2014). PSTv-51 is a new race that combines virulence previously observed in PST-114 and PST-127

^b^First year the original races were identified

^c^Wheat control susceptible to *Pst*

^d^Barley control susceptible to *Psh*

The adult plant *Pst* resistance reactions observed in the field trials in China and USA were consistent with the resistance responses observed for the same lines at the seedling tests under controlled environmental conditions. In all field trials, PI 466050 and PI 466186 were resistant to the field races of *Pst* (chlorotic/necrotic responses with no or slight sporulation, IT scores = 1–4), whereas PI 264220 and PI 560559 were susceptible (abundant sporulation, IT scores = 7–8).

### Identification of a major *Pst* resistance locus on chromosome arm 7HL

In the first evaluation of POP366 and POP371 at SDAU in 2011 both populations showed segregation for responses to *Pst*, with IT scores ranging from 1 to 7. Seeds were obtained from each F_2_ plant and progeny tests were performed for all F_3_ families in 2012 in the same location. Among the 127 F_3_ families analyzed from POP366, 35 were uniformly resistant to *Pst*, 58 showed segregation and 34 were uniformly susceptible, suggesting segregation for a single genetic locus (*χ*^2^_1:2:1_ = 0.97, *P* = 0.62). Among the 131 F_3_ families analyzed from POP371, 33 were homozygous resistant to *Pst*, 67 showed segregation and 31 were homozygous susceptible, also suggesting segregation at a single genetic locus (*χ*^2^_1:2:1_ = 0.13, *P* = 0.94).

**Fig. 1:**
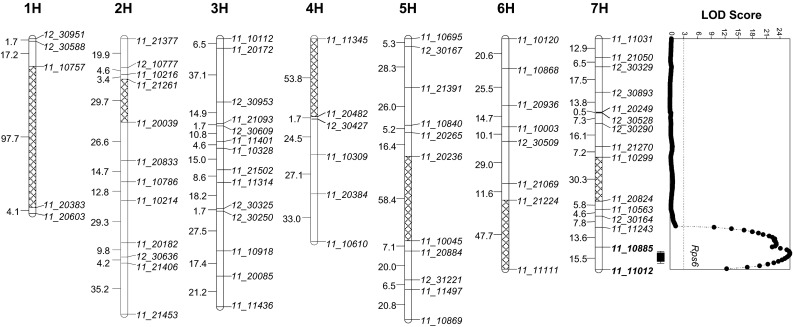
Linkage groups and QTL for *Puccinia striiformis* f. sp. *tritici* (*Pst*) resistance in POP371. A total of 69 polymorphic SNP were initially mapped into 11 linkage groups that were integrated into the 7 barley chromosomes using information from a previous map (Comadran et al. 2012) and from barley genome assembly 082214v1. Cross-hatched regions indicate gaps in our linkage data inferred from the published data. QTL analysis identified a single significant QTL on the distal region of chromosome 7HL that is presented to the right of that chromosome

To map this resistance locus, we genotyped 93 F_2_ plants from POP371 and the two parental lines using the 384-SNP Illumina VeraCode custom assay described in the “Materials and methods”. We identified 71 polymorphic markers and were able to map 69 of them to 11 linkage groups with a cumulative map distance of 831.2 cM (two SNPs remained ungrouped). Using a published genetic map of barley 9K iSelect chip (Comadran et al. 2012), we assigned the mapped markers to their known chromosome locations and generated an integrated genetic map including seven linkage groups (Fig. 1).

A QTL analysis of the *Pst* resistance scores obtained in the F_2_ plants in 2011 revealed a single significant QTL between markers *11_10885* and *11_11012* on the long arm of chromosome 7H. This QTL was associated with a LOD score of 25.8 and explained 30.5 % of the phenotypic variation in *Pst* resistance. No other QTL with a LOD score higher than three was identified, suggesting the presence of a single major *Pst* resistance locus segregating in POP371. However, we cannot rule out the possibility of additional QTL in regions not covered by this map.

**Fig. 2:**
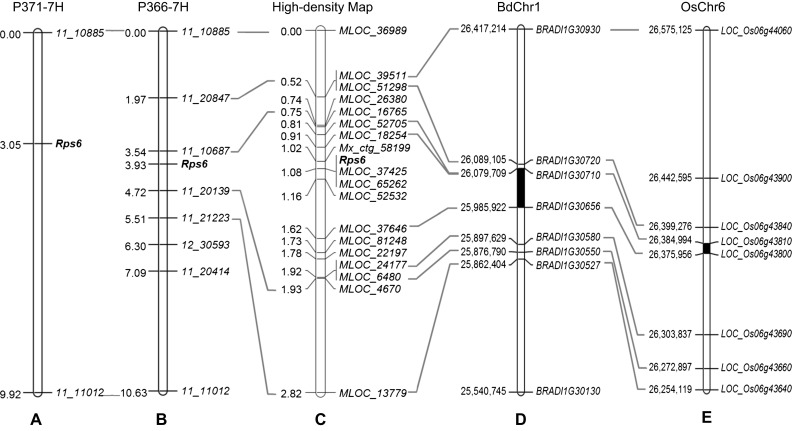
Genetic maps of the *Rps6* region and their colinearity with *Brachypodium* and rice sequenced genomes. **a** Barley genetic map based on POP371 (cM). **b** Barley genetic map based on POP366. **c** Barley high-density map based on 10,888 gametes. **d** Colinear region in *Brachypodium* chromosome 1 (pseudomolecule in bp). **e** Colinear region in rice chromosome 6 (pseudomolecule in bp). *Black* regions in **d** and **e** indicate the *Rps6* candidate region. MLOC numbers are gene identification numbers in Ensembl Plants

We then developed PCR markers for *11_10885* and *11_11012* (Table 2), and used them to map the *Pst* resistance locus in the complete POP371 and POP366 populations. Using the F_3_ progeny test performed in 2012, we mapped *Pst* resistance as a simple Mendelian locus in a similar location in both populations (Fig. 2a, b). These results suggest that resistance to *Pst* in POP366 and POP371 is determined by a major locus that maps 3.0–3.9 cM distal to marker *11_10885* and 6.7–6.9 cM proximal to marker *11_11012* (Fig. 2a, b). Following barley rules for resistance gene nomenclature, this locus has been assigned the formal name *Rps6*.

Using the complete F_2_ population POP371 we estimated the average IT for the plants homozygous for the markers flanking the resistant allele (average IT = 2.2), for the heterozygous plants (average IT = 3.7), and for the plants homozygous for the markers flanking the susceptible allele (average IT = 7). The average IT score of the heterozygous plants was lower (more resistant) than the midpoint between the homozygous resistant and homozygous susceptible plants (IT = (2.2 + 7.0)/2 = 4.6). The degree of dominance of the resistant allele was estimated to be 38 % using the formula described in the “Materials and methods”.

### *Rps6* is allelic to *YrpstY1*

A review of previous studies showed that barley *Pst* resistance gene *YrpstY1* from the Chinese barley line Y12 was mapped on a chromosome region similar to the one identified in this study for *Rps6*. *YrpstY1* was mapped 27 cM from the most distal markers on chromosome arm 7HL (Sui et al. 2010) while *Rps6* was mapped approximately 20 cM from the most distal markers on the same chromosome arm: ~7 cM from *Rps6* to *11_11012* (Fig. 2a) plus 13 cM from *11_11012* to *11_20170* (Barley, OPA 2011, Consensus http://wheat.pw.usda.gov/GG3/maps-short, Muñoz-Amatriaín et al. 2011).

To determine if *Rps6* and *YrpstY1* are allelic we generated reciprocal crosses between resistant lines PI 466050 and Y12 (both carrying a single dominant *Pst* resistance gene), and evaluated the F_1_ and F_2_ plants derived from these crosses for resistance to *Pst* in the UCD field in 2015. PI 466050, Y12 and the two reciprocal F_1_ hybrids were resistant to *Pst* (IT: 0–2). The 260 F_2_ progeny from the cross PI 466050 × Y12 and the 130 F_2_ progeny from the cross Y12 × PI 466050 were all resistant to *Pst* (IT: 0–3), confirming allelism between *Rps6* and *YrpstY1*. Therefore, the low-density map of *YrpstY1* (Sui et al. 2010) should be considered the first map of *Rps6*.

### The *Rps6* locus is also associated with *Pst* resistance in cultivated barley

The previous allelism test suggested that the *Rps6* locus identified in this study in *H. vulgare* ssp. *spontaneum* and the *YrpstY1* identified in *H. vulgare* cultivar Y12 (Sui et al. 2010) are likely alleles of the same gene. This result suggested that *Rps6* is likely to be present in other *H. vulgare* cultivars.

**Fig. 3:**
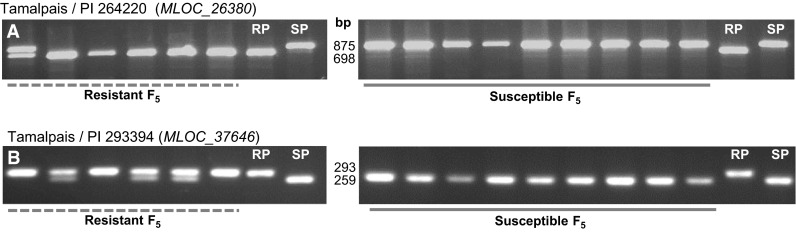
Genotypes of selected F_5_ plants derived from crosses between *Pst* resistant cultivated barley variety Tamalpais and *Pst* susceptible wild barley accessions PI 264220 and PI 293394. **a** F_5_ plants from Tamalpais/PI 264220 genotyped with CAP marker *MLOC_26380* digested with *Taq*I. **b** F_5_ plants from Tamalpais/PI 293394 genotyped with distal marker *MLOC_37646* digested with *Hinf*I. RP = resistant parent (Tamalpais), and SP = susceptible parent (PI 264220 in **a** and PI 293394 in **b**). The size of the amplification products in bp is indicated between the *left* and *right* panels. Markers used for these two loci are based on different SNP than the ones used in the wild barley populations, and their specific primers are listed in Table 2

To test this hypothesis we selected the barley cultivar ‘Tamalpais’ (PI 645477), which displays excellent resistance (IT scores 0–1) to wheat stripe rust in China and USA and crossed it with the susceptible wild barley accessions PI 264220 and PI 293394 (Table 1). Of the 161 F_5_ plants, 42 were susceptible to *Pst* races CYR29, CYR31, CYR32 and CYR33. Since F_5_ plants are 93.75 % homozygous, the observed 3–1 segregation (*χ*^2^_3:1_ = 0.10, *P* = 0.75) is consistent with the hypothesis of segregation for two major resistance genes. We genotyped 20 susceptible and 21 plants with the highest levels of resistance from these two populations using markers *MLOC_26380* (for the Tamalpais/PI 264220 population, Fig. 3a) and *MLOC_37646* (for the Tamalpais/PI 293394 population, Fig. 3b). We found that all susceptible plants (and none of the resistant plants) were homozygous for the susceptible parent allele, confirming that *Rps6* plays an important role in ‘Tamalpais’ resistance to *Pst*. As expected from the selection of the most resistant plants for genotyping, the proportion of plants homozygous for the resistant allele were higher than expected by chance (Fig. 3a, b).

### *Rps6* maps to a 0.14 cM interval between markers *Morex contig_58199* (*Mx_ctg_58199*) and *MLOC_52532*

First, we selected six SNP evenly distributed between markers *11_10885* and *11_11012* from the 9 k iSelect array map (Comadran et al. 2012) (Table 2) and developed PCR markers. We then incorporated these markers in the low-density map from POP366 (127 F_3_ progenies, Fig. 2b) and reduced the *Rps6* candidate region to a 1.2 cM interval between markers *11_10687* and *11_20139* (Fig. 2b).

To develop a high-density map we genotyped 5444 F_3_ plants and identified 746 plants with recombination events between *Rps6* flanking markers *11_10885* and *11_11012* (Fig. 2c). Among the selected plants, we focused on the 129 F_3_ plants that showed recombination events between the closest *Rps6* markers *11_10687* and *11_20139*. These recombinant chromosomes were in heterozygous state and segregated in the progeny tests for *Pst* resistance performed at the UCD field in 2015 (F_4_ plants). Using this information we mapped the *Rps6* locus 0.33 cM distal to *11_10687* (= MLOC_16765) and 0.85 cM proximal to *11_20139* (= MLOC_4670, Fig. 2c). These genetic distances were very similar to the ones obtained in the low-density map of POP366 (Fig. 2b).

To map the recombination events more precisely, we generated additional markers in the *11_10687*–*11_20139* interval using sequence information from barley (http://webblast.ipk-gatersleben.de/barley/viroblast.php), and the colinear regions in the genomes of *A. tauschii* (http://avena.pw.usda.gov/wheatD) (Luo et al. 2013), *Brachypodium* (http://www.plantgdb.org/BdGDB) and rice (http://rice.plantbiology.msu.edu/cgi-bin/gbrowse/rice/) (Fig. 2c). Using these new markers and available recombination events we mapped *Rps6* completely linked to markers *MLOC_37425* and *MLOC_65262*, and within a 0.14 cM region flanked in the distal side by *Mx_ctg_58199* (0.06 cM) and in the proximal side by *MLOC_52532* (0.08 cM, Fig. 2c).

**Fig. 4:**
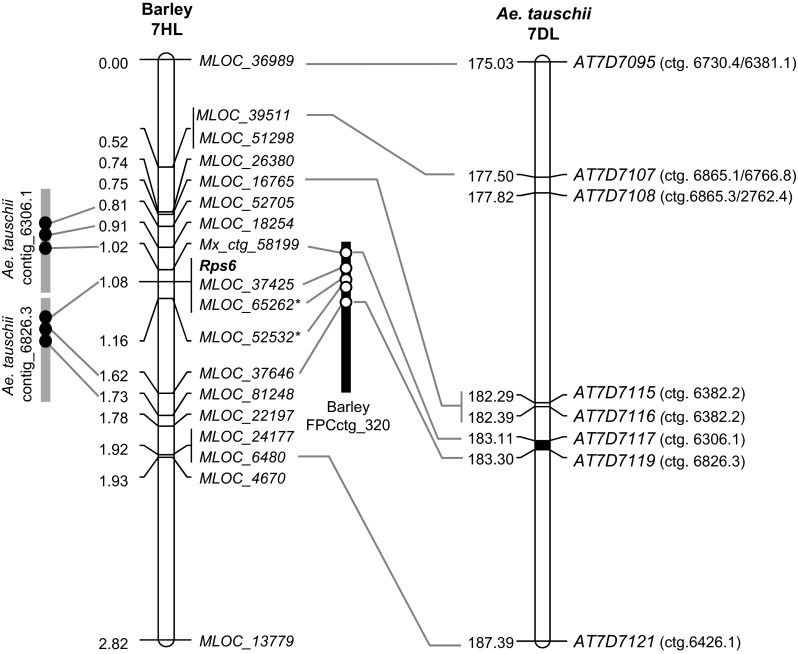
Comparison between the high-density map of *Rps6* in barley chromosome 7HL and the colinear regions in the genetic map and contig sequences of *Aegilops tauschii* chromosome 7DL, and the FPC physical maps of barley. The region in *black* in the genetic maps indicates the candidate region for *Rps6*. The barley and *Aegilops tauschii* contigs are just schematic representations and are not at scale. *Asterisks* after *MOLC_65262* and *MOLC_52532* indicate that these are CC-NBS-LRR genes

BLASTN searches of the sequences from the markers in the *Rps6* region against the IPK-Gatersleben database of BAC end-sequences showed that markers *Mx_ctg_58199*, *MLOC_37425*, *MLOC_65262*, *MLOC_52532*, and *MLOC_37646* have perfect matches (100 %, >500 bp) to BACs located in the large FPC contig_320 (3.46 Mb). The order of the markers in the high-density map was colinear with the order of the corresponding BACs in FPC contig_320 (Fig. 4). The BACs with BAC end-sequences matching markers *Mx_ctg_58199* (HVVMRXALLeA0155A06) and *MLOC_52532* (HVVMRXALLHB00096P12) delimit a region of 501 kb in FPC contig_320.

### The *Rps6* target region in barley is colinear with regions in the *Brachypodium* and rice genomes

Since the complete sequence of FPC ctg_320 is still not available, we explored the colinear regions from other sequenced genomes to search for potential candidate genes. Of the eighteen markers incorporated on the barley high-density genetic map (Fig. 2c), eight are perfectly colinear with *Brachypodium* chromosome 1 and rice chromosome 6 (Fig. 2c–e). These data indicate good conservation of gene order in this region among barley, *Brachypodium*, and rice genomes.

Since no orthologs were found in the *Brachypodium* and rice colinear regions for the closest barley markers flanking *Rps6* (*Mx_ctg_58199* and *MLOC_52532*), we used the next adjacent markers *MLOC_18254* and *MLOC_37646* (Fig. 2c) to determine the colinear candidate region in these two model plant species. These two markers are located 0.7 cM apart in the high-density map and their orthologs define a 93.8 kb in *Brachypodium* chromosome 1 (25,985,922 to 26,079,709) and a 9 kb region in rice chromosome 6 (26,375,956–26,384,994).

The colinear region in *Brachypodium* contains five genes between the two flanking markers (Fig. 2d; Table 4) (http://www.plantgdb.org/BdGDB/). Three of these five genes are predicted proteins of unknown function, while the other two are annotated as a *Cytochrome P450 71D8*-*like* (*Bradi1g30700*) and a predicted *Zinc finger MYM*-*type protein 1*-*like* (*Bradi1g30672*). We were not able to find barley orthologs for any of these five *Brachypodium* genes in the 7HL target region. The colinear region in rice contained no additional genes between the rice orthologs of the barley flanking markers (*LOC_Os06g43800* and *LOC_Os06g43810*, http://rice.plantbiology.msu.edu/cgi-bin/gbrowse/rice/).

**Table 4:** *Brachypodium* genes in the region colinear to the *Rps6* candidate region

Gene name in *Brachypodium*	Barley locus	Predicted function
*Bradi1g30710.1*	*MLOC_52705* ^a^	Uncharacterized protein
*Bradi1g30700.1*	Not found	Cytochrome P450 71D8-like
*Bradi1g30690.1*	Not found	Uncharacterized protein
*Bradi1g30680.1*	Not found	Uncharacterized protein
*Bradi1g30672.1*	Not found	Zinc finger MYM-type protein 1-like
*Bradi1g30664.1*	Not found	Uncharacterized protein
*Bradi1g30656.1*	*MLOC_37636* ^a^	Methyltransferase chloroplastic-like

^a^Flanking markers outside the *Rps6* candidate region

Since no promising candidate genes were identified in the *Brachypodium* or rice colinear regions, we searched for additional candidate genes in the colinear regions in *A. tauschii* (http://aegilops.wheat.ucdavis.edu/ATGSP/blast.php). Six barley markers showed significant sequence identity with sequence flanking five SNPs spanning the region between 175.0 and 187.4 cM on *A. tauschii* chromosome 7D, and good colinearity was detected between these markers (Fig. 4).

The barley markers flanking *Rps6* showed significant similarity to *A. tauschii* contigs_6306.1 and 6826.3, designated hereafter as Aet_ctg_6306.1 and Aet_ctg_6826.3. The first sequenced contig (Aet_ctg_6306.1) is 322.7 kb long and shows significant similarity with proximal barley loci *MLOC_52705*, *MLOC_18254*, and *Mx_ctg_58199* (Fig. 4). The second sequenced contig (Aet_ctg_6826.3) is 257 kb long and shows significant similarity with *Rps6* linked locus *MLOC_37425* and with distal locus *MLOC_37646* (Fig. 4). The annotation of the sequences of these two D genome contigs showed no additional genes in Aet_ctg_6306.1, and one additional gene in Aet_ctg_6826.3, which was annotated as a ribonuclease 3-like protein 2.

### Expression of genes linked to *Rps6*

**Fig. 5:**
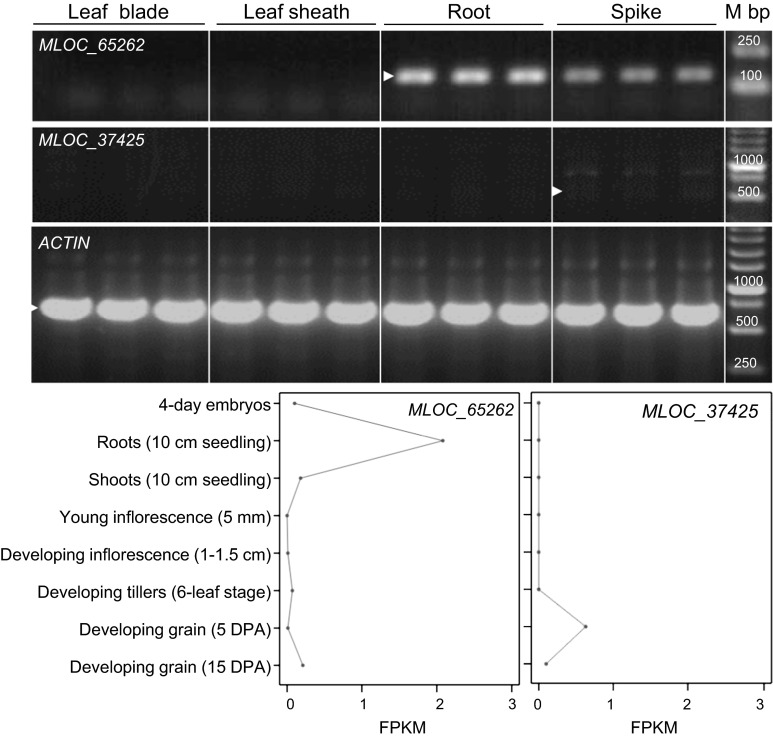
Expression profiles of *MOLC_65262* and *MOLC_37425*. The *top panel* shows expression of the two genes in RNA samples extracted from leaves (blades and sheaths) and roots from Golden Promise plants at the three-leaf stage and from spikes before anthesis. *ACTIN* was used as endogenous control. The *white arrowheads* indicate the expected size based on coding sequence. The *lower panels* are *MOLC_65262* and *MOLC_37425* RNAseq results for eight tissues from the BARLEX database (Colmsee et al. 2015). Expression levels are presented as fragments per kb per million reads mapped (FPKM)

Analysis of the expression of *MLOC_37425* and *MLOC_65262* in leaves (blades and sheaths), roots, and spikes of the variety Golden Promise showed that *MLOC_37425* was expressed only in the spikes at very low levels, whereas *MLOC_65262* was expressed mainly in roots and spikes (Fig. 5). None of these genes was detected in the RNA samples collected from leaf blades and sheaths. Analysis of RNAseq data for eight tissues available in BARLEX (Colmsee et al. 2015) showed similar expression profiles. *MLOC_37425* was not detected in leaves or roots and was expressed at low levels in early grain development. The wheat homolog of *MLOC_37425* (Traes_7BL_DA7413B04.1, http://wheat.pw.usda.gov/WheatExp/) was also expressed during spike development and early grain development but not in the leaves (data not shown). In the BARLEX database, *MLOC_65262* showed expression in roots but not in leaves (Fig. 5).

## Discussion

### Delimitation of the *Rps6* candidate gene region in different grass species

Using orthologs of *Rps6* flanking markers we delimited colinear target regions in rice (<9 kb), *Brachypodium* (<94 kb), and *A. tauschii* (0.2 cM, Fig. 4). In the colinear target region in rice chromosome 6, no additional gene was detected (Fig. 2e). In the colinear target region in *Brachypodium* chromosome 1, five putative genes were detected, but none of them have barley orthologs on the target sequence of chromosome arm 7HL (Fig. 2d; Table 4). In *A. tauschii* contig Aet_ctg_6826.3 we found one additional gene similar to barley *MLOC_81248*, which was annotated as a ribonuclease 3-like protein 2. However, *MLOC_81248* was mapped between *MLOC_37646* and *MLOC_22197*, outside the *Rps6* candidate region (Fig. 4). The colinear target region in *A. tauschii* was estimated to be less than 0.2 cM long (between 183.1 and 183.3 cM), and most of the markers were found in two large sequenced contigs (Fig. 4). However, there is still a gap between the two *A. tauschii* contigs in this region, so we cannot rule out the presence of additional genes in the *Rps6* colinear regions in this species.

The analysis of the colinear regions in rice, *Brachypodium* and *A. tauschii* provided useful information about the similarities and differences among these orthologous regions, but did not identify promising candidate gene for *Rps6*. Therefore, a dedicated effort in barley will be required to identify *Rps6*. As a first step to the positional cloning of this gene we developed a high-density genetic map and delimited the *Rps6* target region to a small genetic interval of 0.14 cM, which corresponds to a ~500 kb region within FPC contig_320 from barley cultivar Morex (Fig. 4).

Once the sequence of this region of chromosome arm 7HL becomes available, it will be possible to develop additional markers and further dissect the *Rps6* target region. We have already identified 15 plants with recombination events between *Rps6* and flanking markers *Mx_ctg_58199* and *MLOC_52532*, which can be used to map these additional markers closer to *Rps6*, narrowing the candidate gene region.

Barley genes *MLOC_37425* and *MLOC_65262* were mapped completely linked to *Rps6* and were considered initially as potential candidate genes. *MLOC_37425* encodes a poorly annotated protein that includes a Myb-like DNA-binding domain (pfam00249) that is expressed at low levels in the spikes and early grain development (Fig. 5). *MLOC_65262* encodes a CC-NBS-LRR resistance gene that was detected only in the roots and spikes. The lack of expression of these two genes in the leaves, where *Pst* resistance is expressed, suggests that *MLOC_37425* and *MLOC_65262* are not good candidate genes for *Rps6*.

It is interesting to mention that flanking marker *MLOC_52532* (mapped only 0.08 cM distal to *Rps6*) is also a CC-NBS-LRR resistance gene (Fig. 2c). Since NBS-LRR genes are frequently present in clusters including multiple resistance genes, we cannot rule out the possibility that additional NBS-LRR genes may be present in the un-sequenced part of the *Rps6* candidate region. In addition, we currently do not known if *Rps6* is present in the barley variety Morex. If *Rps6* is deleted in Morex, additional studies in barley varieties carrying this gene will be necessary to clone *Rps6*.

### Relationship between *Rps6* and other barley resistance genes conferring resistance to different *P. striiformis* formae speciales

In addition to the *Rps6* locus on chromosome arm 7HL, previous studies have identified other barley loci that confer resistance to different *P. striiformis* formae speciales. Pahalawatta and Chen (2005) identified two loci in the barley variety Steptoe that confer resistance to *Pst* races PST-41 and PST-45, and designated them as *RpstS1* and *rpstS2* (Pahalawatta and Chen 2005). The dominant *RpstS1* locus was mapped on chromosome 4H between resistance gene analog polymorphism (RGAP) markers M1 and M2. The second *Pst* resistance locus from Steptoe, *rpstS2*, was not mapped, but its recessive nature suggests that is different from *Rps6*.

Two other loci conferring resistance to *P. striiformis* f. sp. *pseudo*-*hordei* (barley grass yellow rust = *Bgyr*) have been mapped on the long arm of barley chromosome 7H (Derevnina et al. 2015; Golegaonkar et al. 2013). The first one, designated as *Rpsp*-*hYerong*, confers a dominant resistance to *Bgyr* isolate 981549, and was mapped tightly linked to DArT marker *bPb*-*6167* (Derevnina et al. 2015). Marker *bPb*-*6167* and *Rps6* are both located 7 cM proximal to SNP marker *11_1 1012* (Fig. 2), suggesting that *Rpsp*-*hYerong* and *Rps6* are close to each other. This is also supported by the conclusion of Derevnina et al. (2015) that *Rpsp*-*hYerong* is located less than 2 cM from *YrpstY1*, which was shown in this study to be allelic to *Rps6*. An allelism test, or a high-density map of *Rpsp*-*hYerong*, will be necessary to determine if *Rpsp*-*hYerong* is a different gene or if it is allelic to *Rps6/YrpstY1*. A field study of the Yerong/Franklin double haploid population performed in CIMMYT (Toluca, Mexico) showed that the most significant marker for *Bgyr* resistance (DArT marker *bPb*-*6167*) was also the most significant marker for resistance to *Psh*. This result suggests that the *Rpsp*-*hYerong* locus is associated with resistance to two different *P. striiformis* formae speciales. If future allelism studies confirm that *Rpsp*-*hYerong* and *Rps6* represent the same locus, this will indicate that the underlying gene is effective against three different *P. striiformis* formae speciales. The broad spectrum of resistance conferred by this gene makes it a valuable target for positional cloning.

An additional recessive seedling resistance locus against *Bgyr* was detected in the barley variety ‘Sahara 3771’ and was temporarily designated as *rpsSa3771* (=*Bgyr1*) (Golegaonkar et al. 2013). This locus was mapped on chromosome arm 7HL, 13 cM proximal to marker *wg420*, which is closely linked to *bPb*-*6167* (0.9 cM, *Hordeum*-Consensus2006-DArT map). Based on these map comparisons, *rpsSa3771* seems to map roughly 12 cM proximal to *Rps6*, suggesting that they are different genes. This hypothesis is also supported by differences in infection reactions to *Bgyr* isolate 981549 (Derevnina et al. 2015) and in the degree of dominance between these two loci. Resistance against this particular isolate is recessive for *rpsSa3771* and dominant for *Rpsp*-*hYerong* (Derevnina et al. 2015). *Rps6* also showed partially dominant resistance to *Pst* in the experiments described in this study. Taken together, these results suggest that *rpsSa3771* and *Rps6* are different resistance genes.

### Intermediate host resistance

The previous results indicate that at least three different loci (*Rps6*, *RpstS1* and *rpstS2*) can contribute to barley intermediate host resistance to *Pst*. So far, *Pst* resistance genes *RpstS1* and *rpstS2* have been reported only in the cultivated barley variety Steptoe. In contrast, *Rps6* appears to be more widely distributed, since it was detected in the two *Pst* resistant wild barley accessions characterized in this study and in the cultivated variety Tamalpais. Based on the allelism test with *YrpstY1*, *Rps6* seems to be also present in the Y12 barley accession from China (Sui et al. 2010) and in the cultivated variety Abed Binder 12 reported in the companion study (Dawson et al. 2016). However, we recognize that the number of genetics studies of *Pst* resistance in barley is still too small to make a valid generalization about the frequency of the different alleles.

In general, wheat stripe rust shows low levels of infection on barley and does not cause significant damage to barley crops. However, if barley resistance to *Pst* is determined by a limited number of resistance genes, exceptions to this general pattern are expected. Among the 32 *H. vulgare* ssp. *spontaneum* accessions evaluated in the UCD field in 2010, three showed susceptibility to *Pst* (9.4 %, Table 1), which was later confirmed in controlled inoculations. The frequency of *Pst* susceptibility in cultivated barley seems to be lower based on the observation that only a few cultivated barley varieties were reported to be susceptible to *Pst*. However, a detailed study of six barley varieties from Canada with 38 *Pst* isolates showed that three varieties were resistant to all *Pst* races, whereas each of the other three, showed susceptibility to 2, 32 and 36 *Pst* races, respectively (Kumar et al. 2012). This suggests that particular sets of barley accessions may have relatively high frequencies of susceptibility to *Pst* or that some *Pst* races are particularly virulent on barley resistance genes. Broader studies including diverse barley germplasm collections and multiple *Pst* races will be required to answer the previous questions. It will be also interesting to investigate if cultivated barley lines from different geographic origins have similar or different *Pst* resistance genes.

Results from this and previous studies suggest that barley intermediate host resistance to *Pst* depends in many cases on few major resistance genes, and that the difference between host and intermediate host resistance between wheat and barley may be more tenuous than previously thought. The limited time since the divergence between wheat and barley [~11 million years (Huang et al. 2002)] might have been insufficient for the development of a more robust nonhost resistance system. As expected, the close evolutionary relation between wheat and barley seems to be also reflected in the relationship between their respective *P. striiformis* pathogens. A study using Random Amplified Polymorphic DNA markers (RAPD) showed that formae speciales *Pst* and *Psh* are more closely related to each other than to *P. striiformis* f. s. *poae* (Chen et al. 1995). A study using morphological evidence and nuclear rRNA internal transcribed spacer and β-*tubulin* sequences also concluded that *Pst* and *Psh* were more related to each other than to *Puccinia* samples collected from species of *Poa*, *Dactylis* or *Achnatherum* (Liu and Hambleton 2010). The initial RAPD (Chen et al. 1995) and isozymes studies (Newton et al. 1985) suggested that *Pst* and *Psh* are well differentiated groups. However, the more recent studies based on nuclear rRNA internal transcribed spacer and β-*tubulin* sequences suggest more complex relationships (Liu and Hambleton 2010).

In summary, results from this study suggest that barley resistance to *Pst* is not effective in all barley accessions and is determined by a simple genetic basis, supporting its classification as intermediate host resistance. These observations also support the hypothesis that effectiveness and genetic complexity of nonhost resistance is correlated with the degree of evolutionary divergence between the host and nonhost plant species (Bettgenhaeuser et al. 2014).

### Potential applications of nonhost resistance to crop improvement

The use of intermediate host resistance genes against *P. striiformis* can benefit both barley and wheat. Barley genes conferring resistance to *Pst* can be used to improve wheat resistance against *Pst*, whereas wheat genes conferring resistance to *Psh* can be used to improve barley resistance to *Psh*. In this study we focused on the precise mapping of the barley *Pst* resistance locus *Rps6* with the long-term objective of cloning this gene and use it as a potential source of *Pst* resistance for wheat.

This study shows that *Rps6* is effective against all *Pst* races tested so far from China and North America. The resistance to North American race PSTv-51 is particularly important, because this race is virulent to all 18 *Pst* resistance genes in the wheat differential set, except *Yr5* and *Yr15* (Table 3). Race PSTv-51 combines virulences present in previous races PST-114 and PST-127, which represent the two major *Pst* groups detected in the Western US in recent years (Chen et al. 2010; Wan and Chen 2012). In the companion paper, *Rps6* was also shown to be effective against *Pst* races from the UK (Dawson et al. 2016). *Rps6* broad spectrum resistance to *Pst* suggests that this gene may be a valuable tool to control stripe rust in wheat.

Another example of successful use of a nonhost resistance gene among grass species was the transfer of the maize nonhost resistance gene *Rxo1* to rice. Rice plants transformed with this maize gene were resistant against *Xanthomonas oryzae* pv. *oryzae*, a pathogen that causes bacterial streak disease in rice (Zhao et al. 2005). A similar transgenic strategy can be used to introgress *Rps6* into wheat, once the gene is identified in barley. However, it could also be possible to transfer this gene to wheat by homoeologous recombination, avoiding the costly regulatory processes associated with the release of transgenic commercial varieties. An addition line of chromosome 7H from barley variety ‘Betzes’ into wheat cultivar ‘Chinese Spring’ (Islam et al. 1981), and a spontaneous translocation between chromosome arms 7HL from barley variety ‘Manas’ and 4BS from wheat variety ‘Asakaze Komugi’ (Cseh et al. 2011) are available. If *Rps6* is present, the 4BS·7HL translocation would be a better source than the 7H addition line to transfer the distal region of 7HL into wheat by homoeologous recombination.

However, the transfer of *Rps6* to wheat by homoeologous recombination faces several challenges. The first challenge is the low recombination rate observed between barley and wheat chromosomes even in the absence of the *ph1b* gene (Islam and Shepherd 1992). The second challenge is the potential transfer of undesirable linked traits. It is known that the *Phytoene synthase 1* (*PSY*-*1*) gene located in the distal end of the long arm of group 7 is associated with the presence of yellow pigments in the flour (Rodriguez-Suarez and Atienza 2012; Zhang and Dubcovsky 2008). If present, this negative effect can be separated from *Rps6* by a second round of homoeologous recombination or by mutagenesis, as done before for the *PSY*-*E1* gene present in the 7EL translocations from *Lophopyrum elongatum* (Zhang and Dubcovsky 2008).

The high-density map and the molecular markers developed in this study provide the information and tools required to accelerate the transfer of *Rps6* into wheat. If the homoeologous recombination strategy is selected, the markers and maps developed here can be used to monitor and select recombination events close to the *Rps6* region. If a transgenic strategy is selected, the two completely linked markers and the closest flanking markers can be used as starting points for the positional cloning of *Rps6*. The broad resistance conferred by *Rps6* to all *Pst* races tested so far (and possibility to some races of *Psh* and *Bgyr*) justifies the effort.

#### Author contribution statement

DF and JD designed the research. KL preformed research and coordinated the experimental part of the project. JH, CZ, AW, JW, GBG, and XC performed research. KL, JH, JD, MMA, and DF analyzed data. KL wrote the first version of the manuscript. All authors reviewed the manuscript. JD provided a major revision of the manuscript and integrated the different contributions.
